# Molecular Characterization and Biodiversity of a Putative Chlorotoxin from the Iranian Yellow Scorpion *Odontobuthus doriae*

**DOI:** 10.18869/acadpub.ibj.21.5.342

**Published:** 2017-09

**Authors:** Maryam Naderi Soorki, Amir Jalali, Hamid Galehdari

**Affiliations:** 1 Department of Genetics, Faculty of Science, Shahid Chamran University of Ahvaz, Ahvaz, Iran; 2 Department of Pharmacology and Toxicology, School of Pharmacy and Toxicology Research Center, Ahvaz Jundishapur University of Medical Sciences, Ahvaz, Iran

**Keywords:** Chlorotoxin, Scorpion, *Odontobuthus doriae*, cDNA library

## Abstract

**Background:**

Chloride channels have already been over-expressed in the different types of cancer. Chlorotoxins, as the blocking agent of these channels, have been indicated to be an effective drug against tumors. In this study, we characterized a putative chlorotoxin from a cDNA library of the venom glands obtained from the Iranian scorpion *Odontobuthus doriae*.

**Methods:**

A cDNA library was constructed from venom gland transcriptome of six scorpions. The cDNA encoding *Odontobuthus doriae* chlorotoxin was isolated from the library, and its putative peptide was characterized by some bioinformatics software such as protein blast, SignalP4.0, DISULFIND and Clustal Omega.

**Results:**

The mature *Odontobuthus doriae* chlorotoxin peptide has a 35-amino-acid residue and four disulfide bounds. This putative chlorotoxin is a small, compact, and stable molecule. Moreover, based on the open reading frame sequence similarity, this peptide is similar to *Buthus martensii* Karsch chlorotoxin-like toxin and Bm12-b neurotoxins from the Chinese scorpion *Mesobuthus martensii*.

**Conclusion:**

The small size of this putative chlorotoxin and its stability make it as a suitable candidate for medical and pharmacological research, especially in the cancer research.

## INTRODUCTION

Scorpion venom is a great source of different peptides[1]. Decades ago, scorpion-based peptides were isolated and purified, because these molecules may target various ion channels and cell membrane components[2]. Voltage-gated ion channels in neural cells membrane are usually the scorpion venom targets. These toxins lengthen the action potential and/or repeatedly trigger the neural cells and cause the aggregation of Ca^2+^ or Na^+^ ions inside the cell, which in turn result in insufficient release of neurotransmitters from the influenced tissues[3].

In the recent decades, a number of investigations have been dedicated to diagnosis and treatment of cancer. In spite of the remarkable advancement in cancer treatment, there are still some limitations, including the lack of selectivity, invasive side effects, and insufficient efficacy[4]. A new approach in battling cancer is required to find novel natural compounds with higher selectiveness and fewer side effects.

Anticancer peptides are significant sources for designing the new targeted drugs. Peptides of small sizes can penetrate the tumor cells and destroy them[5,6]. Chlorotoxins are short peptides (about 36 amino acids) with four disulfide bounds. In a study, it has been demonstrated that chlorotoxin isolated from *Leiurus quinquestriatus* venom inhibits the small channels of chlorine extracted from the epithelial cells[7]. Other studies have indicated the attachment of these peptides to the chloride channels of human cells (such as astrocytoma and glioma), which its mechanism is carried out through connection to metalloprotease-2[8,9]. Chlorotoxin-metalloprotease-2 complex is allocated for neuroectodermal cells of glioma and tumor cells. However, chlorotoxins do not attach to the human normal cells[10]. Liposomal improved chlorotoxin has been shown to be able to significantly inhibit 4T1 breast tumor cells (a cell line of metastatic breast cancer), which express a large amount of metaoproteas-2[11].

Considering the nature of the scorpion venom discussed earlier, scorpion peptides are appropriate candidates for the possible generation of natural medications to cure diseases[12,13]. In the present study, for the first time, cDNA sequence of a chlorotoxin in the Iranian scorpion *Odontobuthus doriae* venom gland cDNA library was isolated and analyzed.

## MATERIALS AND METHODS

### The cDNA library construction

Total RNA was extracted from the active venom glands of six *Odontobuthus doriae* scorpions that had been milked three days prior to the RNA extraction (Qiagen® RNeasy Mini Kit, TAKARA Co., UK). The RNA concentration was measured by NanoDrop (Thermo Fisher Co., USA). First-strand and second-strand cDNA synthesis and linker addition were carried out using the In-Fusion® SMARTer® Directional cDNA Library Construction Kit (Takara Bio Inc., Canada).

The quality and the quantity control of cDNA were checked by both 1.2% agarose gels and NanoDrop. Ligation of cDNA into pSMART21F vector and transformation of vectors to chemically competent bacterial cells were done according to the suggested protocol by manufacturer. Transformed cells grown on Luria Bertani agar plate contained 100 µg/ml ampicillin, 1 mM isopropyl-beta-D-thiogalacto-pyranoside, and 75 µg/ml 5-Bromo-4-chloro-3-indolyl β-D-galactopyranoside.

To select the positive colonies, random screening through the blue/white colony selection and colony PCR using flanking PCR primers were performed. The selected PCR fragments corresponded to the expected length of toxin and venom components transcripts (around 500–1000 bp). The plasmid DNA of the selected colonies was extracted by QIAprep Spin Miniprep Kit (TAKARA Co., UK), and the cDNA inserts were sequenced (Macrogen® Co., Korea).

### Bioinformatics analysis

cDNA sequence of chloride toxin was checked by VecScreen tools (http://www.ncbi.nlm.nih.gov/tools/vecscreen/) to trim from vector and primers sequence contaminations. The amino acid sequence of the obtained cDNA sequence was deduced using the ORF Finder software (http://www.ncbi.nlm.nih.gov/projects/gorf/). The sequence of detected ORF was confirmed by protein BLAST NCBI (http://www.ncbi.nlm.nih.gov/). The preparation of phylogenic tree and amino acid alignment were performed using online tools in UniProt website (http://www.uniprot.org/). Signal peptide sequence was predicted by signalP4.1 available at http://www.cbs.dtu.dk/services/SignalP/. Number of disulfide bounds and their positions in sequence were predicted by DISULFIND online tool (http://disulfind.dsi.unifi.it/)[14]. Molecular weight and theoretical isoelectric pH were estimated by ProtParam tool (http://web.expasy.org/protparam/). The molecular modeling of the second and third structure of putative mature peptide was done using online tools in SWISS-MODEL website (http://swissmodel.expasy.org/).

## RESULTS AND DISCUSSION

**Fig. 1:**
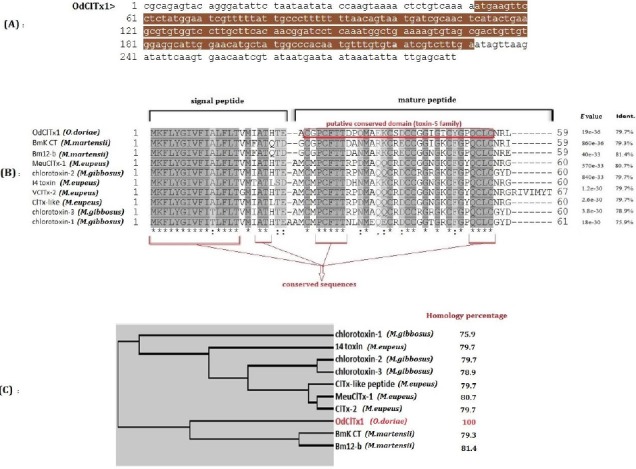
Molecular characterization of OdClTx1. (A) Nucleotide sequence of cDNA-encoding OdClTx1 with its coding DNA sequence region that is shown in brown. (B) Results from alignment of OdClTx1 precursor peptide with homologues peptides from other scorpion species, *Mesobuthus martensii* (*M. martensii*), *Mesobuthus eupeus* (*M. eupeus*), and *Mesobuthus gibbosus* (*M. gibbosus*). Identity (Ident., %) and *E* values are shown on the right sides that correspond to the precursor peptides. High and low similarities are shown by dark and light gray, respectively. Some conserved sequences are marked on the bottom of picture. Putative conserved domain belonging to toxin-5 family is marked with red box. Putative signal peptide and mature peptide are indicated on top of the sequences. (C) Dendrogram of OdClTx1 biodiversity along with other similar chlorotoxins from other species of buthidae family with the percent of homology, which are shown on the right.

In the present study, we isolated a chloride channel toxin (ClTx) from the cDNA library of the venom gland of a medically important scorpion in Iran, *Odontobuthus doriae*. This scorpion belongs to the *Buthidae* scorpion family. The nucleotide sequence of cDNA encoding this putative toxin (named as OdClTx1, hereafter) and the respective peptide sequence were deposited in NCBI Gene Bank database (Gene ID: KU365857.1) (Fig. 1A). Sequence similarity analysis revealed that the OdClTx1 mRNA is similar to the known meu14toxinA mRNA (KU577533.1) with 96% coverage and 86% identity, by the highest confidence. The meu14toxinA was belonged to another Iranian scorpion, namely *Mesobuthus eupeus*. The ORF of the OdClTx1 precursor peptide has 59 amino-acids with the highest similarity score to the *Buthus martensi Karsch* chlorotoxin-like toxin (BmK CT) from *Mesobuthus martensii* by 100% coverage and 80% identity. The OdClTx1 precursor peptide was aligned with nine similar ClTxs from the other scorpions (Fig. 1B).

Putative conserved domain belonging to the toxin-5 superfamily was detected in OdClTx1 putative peptide. This family contains various secreted scorpion toxins that might be unrelated to the pfam00451. The pfam05294 is a member of the superfamily cl05046 from *Buthidae* family. A 24 amino-acid signal peptide was predicted in OdClTx1 precursor peptide.

Biodiversity of the OdClTx1 was examined, and the phylogenic tree of the OdClTx1 with similar peptides was prepared (Fig. 1C). Based on the dendrogram, in scorpion group, the isolated OdClTx1 from the Iranian *Odontobuthus doriae* displayed the highest similarity with the BmK CT and with the Bm-12b neurotoxins of *M. martensii*; both species belong to *Buthidae* family[15]. Therefore, we can conclude that Iranian *Odontobuthus doriae* scorpion has the highest homology with Chinese *M. martensii*.

The BmK CT is a toxin with unknown function in the healthy organisms; however, when tested on gliomas cells, it inhibits chloride currents in a voltage-dependent manner[16]. BmK CT also interacts with matrix metalloproteinase-2 and significantly inhibits its catalytic activity[17]. It may internalize with chloride channels (probably ClC-3/CLCN3) and matrix metalloproteinase-2; thus, inhibiting the chloride channels is necessary for cell shrinkage and inhibition of tumor propagation. This information has provided a valuable resource for further studies on the biology of the OdClTx1 peptide, as well as for future therapeutic approaches, which focus on chloride toxins for human cancers treatment [18]. Further comparison of OdClTx1 with other similar peptides indicated highest and lowest homology with chloride toxins obtained from *Mesobuthus eupeus* and *Mesobuthus gibbous*, respectively. Physico-chemical parameters for 35 amino-acid mature peptide of the OdClTx1 were measured by ProtParam tool. OdClTx1 with “C_148_H_237_N_43_O_48_S_9_” formula has a molecular weight of 3675.3 kDa and a pH of 7.69 in isoelectric point. The residue of N-terminal amino-acid of mature OdClTx1 is known as “Cysteine” that is small, tiny, and hydrophobic. Based on measuring the instability index (21.69), OdClTx1 is a stable molecule. Grand average of hydropathicity (GRAVY) for OdClTx1 has been measured as 0.063; hence, this molecule is a hydrophobe molecule. Due to these parameters, the OdClTx is a small and stable peptide.

Disulfide bridge analysis by predictor servers has been indicated that OdClTx1 has four disulfide bound in positions 1 and 18, 4 and 19, 15 and 30, and 25 and 32. Alignment data showed that the Cysteine residues that participate in these disulfide connections were conserved in similar peptides (Fig. 1B). The presence of numerous disulfide bounds in a small peptide indicates the OdClTx1 as a very small and stable, compacted molecule under the physiologic conditions. Peptides of small sizes can easily permeate the tumor cells and erode them directly or indirectly[5,6]. The ability of natural toxins such as chlorotoxins to establish separate attachments to various cellular domains has created new hopes for the development of the anticancer drugs. Direct attachment of chlorotoxins to chloride channels effectively influence the mechanisms of cancer cell motility and metastatic invasions to the cell [8, 9].

**Fig. 2:**
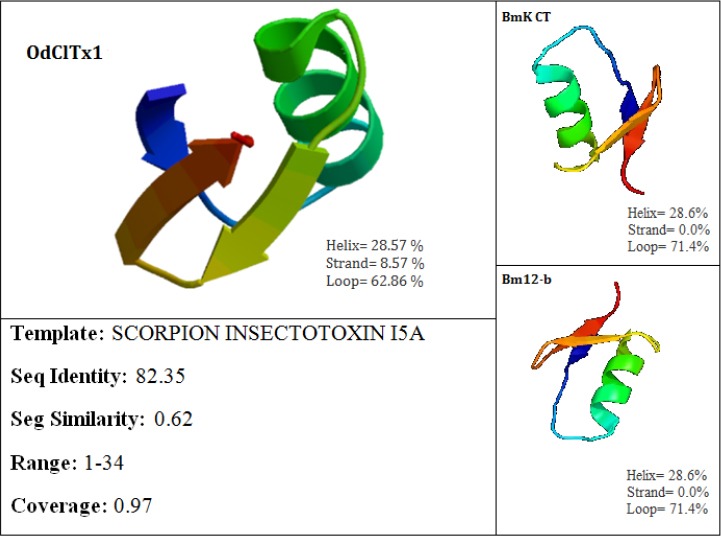
Molecular modeling of OdClTx1. Modeling result of OdClTx1 was predicted from the highest scoring template “scorpion insect toxin 15A” by the “SWISS-MODEL” software (https://swissmodel.expasy.org/). Details from this modeling were obtained by its comparison with two homologue peptides: BmK CT and Bm-12b neurotoxins of *Mesobuthus martensii* (*M. martensii*) (on the right). All of three homologue peptides have the same molecule model predicted from “scorpion insect toxin 15A” by the mentioned software. Image colored by rainbow N → C terminus.

Molecular modeling of mature OdClTx1 along with its details is shown in Figure 2. This model was built with high confidence by the highest scoring template. Based on this model, OdClTx1 mature peptide in three disentail state was more similar to “scorpion insect toxin I5A” with 94% coverage and 82.35% identity. In the built model of OdClTx1, three strand (8.57%), one helix (28.57%), and one loop (62.86%) were found. OdClTx1 folding was done by the highest confidence of similarity with knottin domains. These domains are small inhibitors, toxins, and lectins belonging to the scorpion toxin-like superfamily and short-chain scorpion toxins family[19]. In OdClTx1 mature peptide, the knottins domain was found in positions 1-34-amino-acid sequence (Fig. 2).

For the first time, we isolated a ClTx-encoding cDNA from venom gland cDNA library of the Iranian yellow scorpion *Odontobuthus doriae* and characterized its new peptide as OdClTx1. The homology search of nucleotide and protein sequence of the OdClTx1 in databases confirmed the nature of its toxicity on chloride channels. Due to the high homology of OdClTx1 with BmK CT from the Chinese *M. martensii*, it is possible that OdClTx1 exerts its function by a similar mechanism through the involvement of matrix metalloproteinase-2. As a result of its high stability and small size, OdClTx1 can be considered as a proper candidate for the medical and pharmacological research, especially in cancer area. By preparation of a framework for the expression of the OdClTx1 peptide identified in the current study, we could create a beneficial platform for the future investigations.
